# Lipidomic Signature of Abdominal Aortic Aneurysm and Peripheral Artery Disease

**DOI:** 10.1021/acs.jproteome.5c00293

**Published:** 2025-09-05

**Authors:** Helena Beatriz Ferreira, Tara van Merrienboer, Inês M. S. Guerra, Tânia Melo, Kak Khee Yeung, Venkat Ayyalasomayajula, Fábio Trindade, Rita Nogueira-Ferreira, Adelino Leite-Moreira, Laura Goracci, Rita Ferreira, M. Rosário Domingues, Marina Dias-Neto, VASCUL-AID Consortium

**Affiliations:** † Mass Spectrometry Center, LAQV-REQUIMTE, Department of Chemistry, 56062 University of Aveiro, Campus Universitário de Santiago, 3810-193 Aveiro, Portugal; ‡ Surgery, Amsterdam University Medical Center, University of Amsterdam, Meibergdreef 9, 1105 AZ Amsterdam, The Netherlands; § Physiology, Amsterdam University Medical Center, Vrije Universiteit Amsterdam, De Boelelaan 1117, 1081 HV Amsterdam, Netherlands; ∥ Amsterdam Cardiovascular Sciences, Atherosclerosis & Aortic syndromes, 1105 AZ Amsterdam, The Netherlands; ⊥ CESAM − Centre for Environmental and Marine Studies, Department of Chemistry, 56062 University of Aveiro, Campus Universitário de Santiago 3810-193 Aveiro, Portugal; # UnIC@RISE Department of Surgery and Physiology, 26705 Faculty of Medicine of the University of Porto, 4200-319 Porto, Portugal; ⊗ Department of Cardiothoracic Surgery, Centro Hospitalar Universitário São João, 4200-319 Porto, Portugal; ○ Department of Chemistry, Biology and Biotechnology, 9309 University of Perugia, 06123 Perugia, Italy; ● Department of Angiology and Vascular Surgery, Unidade Local de Saúde São João, 4202-451 Porto, Portugal

**Keywords:** Abdominal aortic aneurysm, peripheral arterial disease, vascular diseases, lipidomics, mass spectrometry

## Abstract

Vascular diseases are powerful predictors of cardiovascular mortality, but they are typically under-recognized and undertreated. There is no effective treatment for either abdominal aortic aneurysm (AAA) or peripheral artery disease (PAD). Lipids are key molecules in cardiovascular diseases and good candidates for diagnosis, monitoring, and risk prediction; nonetheless, there is very limited information on the lipidomic profile of patients with AAA and PAD. We hypothesize that lipids can be used as important prognostic biomarkers of these diseases. To achieve this, we conducted a comprehensive C18 reversed-phase (RP) liquid chromatography-tandem mass spectrometry (LC-MS) lipidomic analysis of plasma from AAA and PAD patients undergoing open repair surgery, comparing their profiles with those of healthy controls. We observed a marked reduction in PAD and AAA of the relative abundances of (i) phospholipids bearing polyunsaturated fatty acids, primarily from the phosphatidylcholine (PC), phosphatidylethanolamine (PE), and phosphatidylinositol (PI) classes, mostly due to oxidative degradation, and (ii) plasmalogen species of PC and PE, which serve as endogenous antioxidants. On the other side, SM and Cer increased in both pathologies. Our findings suggest a dysregulation of the lipid metabolism in AAA and PAD compared with healthy controls that deserves exploration to unravel putative biomarkers or disease hallmarks.

## Graphical abstract

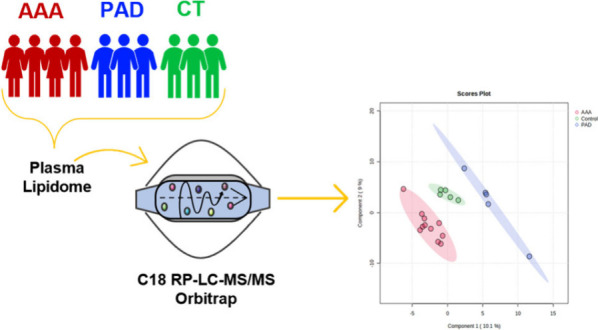

## 1 Introduction

Cardiovascular diseases (CVDs) are responsible for the highest number of deaths worldwide, taking an estimated 20.5 million lives in 2021.^1^ Among those, vascular diseases are powerful and significant predictors of cardiovascular mortality, but they are typically under-recognized and undertreated due to limited screening or silent progression.^2^ Vascular diseases encompass a broad spectrum of conditions, among which abdominal aortic aneurysm (AAA) and peripheral artery disease (PAD) are among the most prevalent and clinically significant, affecting, respectively, 35.12 ^3^ and 110 million^4^ people worldwide.

The characteristic feature of AAA consists of the weakening and dilatation of the abdominal aorta.^5^ AAA is a silent disease, usually asymptomatic, until the major complication of AAA happens, which is the rupture of the aneurysm wall, that commonly leads to fatal bleeding into the retroperitoneum or abdomen, killing between 150,000–200,000 people each year worldwide.^6,7^

Atherosclerosis is a major impact factor for not only AAA but also PAD development. PAD is characterized by stenosis or occlusion of the arteries supplying blood mainly to the lower limbs and is also associated with microvascular dysfunction.^8^ The typical symptom of PAD is intermittent claudication; however, like AAA, most PAD patients do not have symptoms.^9–11^ This vascular disease affects 6% of adults that have an impaired quality of life (those who have symptoms) due to leg pain, walking impairment, and high risk of major adverse cardiovascular events, including amputation and death.^12^

For both vascular diseases, AAA and PAD, there is no effective medical treatment. The best current management of these diseases consists of the aggressive modification of general cardiovascular risk factors. However, it remains unknown which AAA or PAD patients will have cardiovascular events or in which patients the vascular disease will progress. Therefore, it is utterly important to find new prognostic biomarkers that help clinicians to predict not only disease progression but also any future cardiovascular event that the patient may suffer from. High throughput lipidomics is a possible solution to find such biomarkers because it allows the evaluation of the variation of lipids which can be correlated with clinical data for the study of several chronic diseases including, more recently, CVD.^13,14^ For instance, the phospholipids phosphatidylcholine (PC) and phosphatidylethanolamine (PE), and some sphingolipid classes, were found to be strongly related to the outcome of CVD events (CERT2 score). On the other side, polyunsaturated plasmenyl PC and PE lipids were inversely associated with CV outcome.^14^

Lipids are key molecules in the organism and there is evidence of dysregulation in lipid metabolism of patients with AAA and PAD. The circulating lipidome of AAA has been determined to have decreased levels of lysophosphatidylcholine species with triglycerides showing the opposite behavior.^15,16^ In PAD, there is a significant reduction of both esterified and free *n* ^–3^ fatty acids^17,18^ and, on the other side, oxylipins have been reported as having markedly higher levels in these patients.^19,20^ Nonetheless, there is very limited information about the lipidomics profile of patients with AAA and PAD. PAD is missing information on the pathology-induced changes of the lipidome, since the majority of the reports focus on fatty acid analysis, while AAA circulating lipidome remains elusive. Moreover, statins (lipid-lowering drugs) are the first line of treatment for both AAA and PAD patients. Statins inhibit the action of HMG-CoA reductase, which is crucial for cholesterol production, significantly lowering circulating levels of LDL-cholesterol and, consequently, cardiovascular events.^21^ However, besides modifying LDL metabolism, statins have also been found to impact the lipid profile, especially at a phospholipid and sphingolipid level.^22,23^ In this study, we aimed to comprehensively assess the lipid profile from plasma, to find phenotypes, of patients with AAA and PAD that needed open repair surgery (compared to healthy controls), by using an untargeted C18 RP-LC-MS lipidomic approach. As mentioned, AAA and PAD are two vascular diseases that have neither a definite therapy nor a predictive prognosis, and the assessment of their development was left behind during the COVID-19 pandemic. With the prevalence of AAA and PAD expected to increase, identifying prognostic biomarkers could improve patient stratification, early intervention, and better healthcare resource allocation.

## 2 Materials and Methods

### 2.1 Study Population

This was a prospective translational study where plasma samples were obtained from a cohort of consecutive individuals undergoing elective aortic open surgical repair at the Amsterdam University Medical Center, University of Amsterdam, or Dijklander Hospital in Hoorn (The Netherlands) between 2016 and 2024, including patients with abdominal aortic aneurysms (AAAs) and with peripheral artery disease (PAD). Before surgery, informed consent was retrieved from all patients for their blood to be stored in the Biobank for Aortic Aneurysms, Atherosclerosis, and Biomarkers (2017.121). Prior to the incision, blood was drawn into a 6 mL EDTA tube. The filled 6 mL EDTA tube was transported at room temperature to the laboratory. The blood was centrifuged at 3000 rpm for 10 min at room temperature, and the plasma was collected and stored at −80 °C until subsequent lipidomic analysis. Blood from nonpathological controls was carefully selected to ensure age and gender match with the patient groups. Other selection criteria for controls included the overall health status, absence of active infections, AID and immunomodulatory drug treatment, and lipoprotein profiles within the reference range. The number of healthy volunteers included in the study (*n* = 5) is fairly small, particularly in light of the 1:3 ratio compared to patient subgroups. Therefore, we recognize the limited sample size as a constraint of this study. Patient characteristics were obtained from electronic health records and reported as follows (Supplementary Table S1): age, sex, aneurysm size (mm), body mass index (BMI), smoking status, renal dysfunction, previous vascular surgery, antihypertensive drugs use, lipid lowering medication use and antithrombotic drugs use. All patient material was collected according to the Declaration of Helsinki regulations and the institutional guidelines of the Medical Ethical Committee of Amsterdam UMC, located at VU Medical Center. The study protocol was approved by the local Ethics Committee and conducted under the project VASCUL-AID (grant agreement ID: 101080947).

### 2.2 Reagents

The list of reagents and suppliers used in this study is reported in Table S2.

### 2.3 Lipid Extraction

The BUME method was used to extract the lipids from plasma samples.^24^ Briefly, 10 μL of plasma sample was transferred to an eppendorf (prewashed with methanol) to which was added 100 μL of a BUME solution [butanol/methanol, 1:1 v/v, with 10 mM ammonium formate containing 0.5 μg of internal standard PC 40:0 (20:0/20:0) to monitor extraction efficiency. Each eppendorf was vortexed for 10 s and sonicated for 1 h at 18–22 °C. Then, the eppendorfs were centrifuged at 14000*g* for 10 min (at 20 °C). A volume of 69 μL of the supernatant was transferred to a vial with a microinsert.

### 2.4 Lipid Extract Analysis by C18 Reverse-Phase Liquid Chromatography-Mass Spectrometry (RP-LC-MS)

#### 2.4.1 Sample Preparation

A volume of 6 μL of a mixture of internal standards was added to the vials with the microinsert and the 69 μL of sample. The content of the internal standard mixture is reported in the Supporting Information, Table S3. These internal standards were used to determine the relative abundances of the lipid species identified by lipidomic analysis. The initial chromatographic phase used a mixture of two mobile phases: 68% eluent A (60% acetonitrile, 40% methanol, 10 mM ammonium formate, and 0.1% formic acid) and 32% eluent B (90% isopropanol, 10% acetonitrile, 10 mM ammonium formate, and 0.1% formic acid).

#### 2.4.2 Data Analysis and Integration

Lipid separation was performed using a C18 reversed-phase chromatography approach with an Ascentis Express 90 Å C18 HPLC column (15 cm × 2.1 mm; 2.7 μm, Supelco) installed in an HPLC system (Ultimate 3000 Dionex, Thermo Fisher Scientific, Bremen, Germany) equipped with an autosampler. The system was coupled online to a Q-Exactive Hybrid Quadrupole-Orbitrap Mass Spectrometer (Thermo Fisher Scientific, Bremen, Germany). LC-MS/MS settings were defined as previously reported.^25^

LC-MS data were processed using the Lipostar software (version 2.1.4x64)^26^ with a database created from LIPID MAPS structure database (version Octobre 2024). The applied settings for data analysis within the Lipostar software were previously described elsewhere.^27^

#### 2.4.3 Statistical Analysis

Univariate and multivariate statistical analyses were performed using Metaboanalyst 6.0.^28^ The data sets were then normalized to the internal standard, and missing values were replaced by one-fifth of the minimum positive values of their corresponding variables. The data sets were then log transformed (base 10) and autoscaled. Partial Least Squares Discriminant Analysis (PLS-DA) was performed using PERMANOVA. Heatmaps were created using “Euclidean” as the distance measure and “Ward.D” as the clustering method. To test the significance of the differences between conditions, we used *t* test/ANOVA (*p*-values lower than 0.05). The *p*-values were adjusted for multiple comparisons using FDR adjustment (*q*-values).

## 3 Results

### 3.1 Characterization of the Plasma Samples

We conducted a study to assess the changes in the lipid profile associated with AAA and PAD by analyzing plasma samples from AAA (*n* = 11) and PAD (*n* = 5) patients that needed open repair surgery, comparing them with healthy controls (*n* = 5). Within our study cohort, 75% of the patients enrolled were male adults aged between 51 and 85 years old, corroborating the well-established notion that these diseases predominantly affect the male gender. Healthy control volunteers were age and gender matched with at least one AAA or PAD patient to ensure that the observed differences are disease-specific and not a demographic artifact. Both AAA and PAD patients were under standard medical treatment with statins, antithrombotic and antihypertensive agents, while none of them was taking PCSK9 inhibitors (Table S1). It should be noted that the use of lipid-lowering drugs (statins) may influence the lipid metabolism, thus possibly interfering with the plasma lipidome, which will be taken into consideration in the interpretation of the lipidomic data.

### 3.2 Lipid Profile of AAA and PAD Patients

The lipid profiles of plasma samples from AAA, PAD, and Control groups were analyzed using a high-resolution C18 RP-LC-MS/MS platform. This comprehensive lipidomic analysis allowed the identification of 328 distinct lipid species (molecular ions) in the plasma samples (Table S4). These identified species belonged to 16 different lipid classes, namely, fatty acylcarnitine (CAR), phosphatidylcholine (PC) comprising diacyl, alkyl-acyl and alkenyl-acyl species, lyso PC (LPC), phosphatidylethanolamine (PE) including diacyl, alkyl-acyl and alkenyl-acyl species, lyso PE (LPE), phosphatidylinositol (PI), lyso PI (LPI), phosphatidylserine (PS), sphingoid base, ceramide (Cer), hexosylceramide (Hex_n_Cer), sphingomyelin (SM), gangliosides (GM_n_), cholesteryl ester (CE), diacylglycerol (DG), and triacylglycerol (TG). The lipid species from all classes were identified through analysis of exact mass, retention time and MS/MS spectra. Lipid species belonging to the neutral lipid classes DG and TG were not considered to the multivariate and univariate statistical analysis due to their strong link with dietary habits and potential confounding influence.

The plasma lipid profile of AAA and PAD was compared to healthy controls to have a better understanding of the lipid alterations that happen in these pathologies. A multivariate analysis of the data sets was performed, and the PLS-DA plot generated showed a clear separation of the three groups (Figure 1). The PLS-DA score plot described 19.1% of the total variance, including dimension 1 (10.1%) and dimension 2 (9%), where dimension 1 was the major discriminant. AAA samples were scattered in the right region while PAD samples were scattered in the left region of the plot. Control samples were clustered on the middle section of the plot.

**Figure 1:**
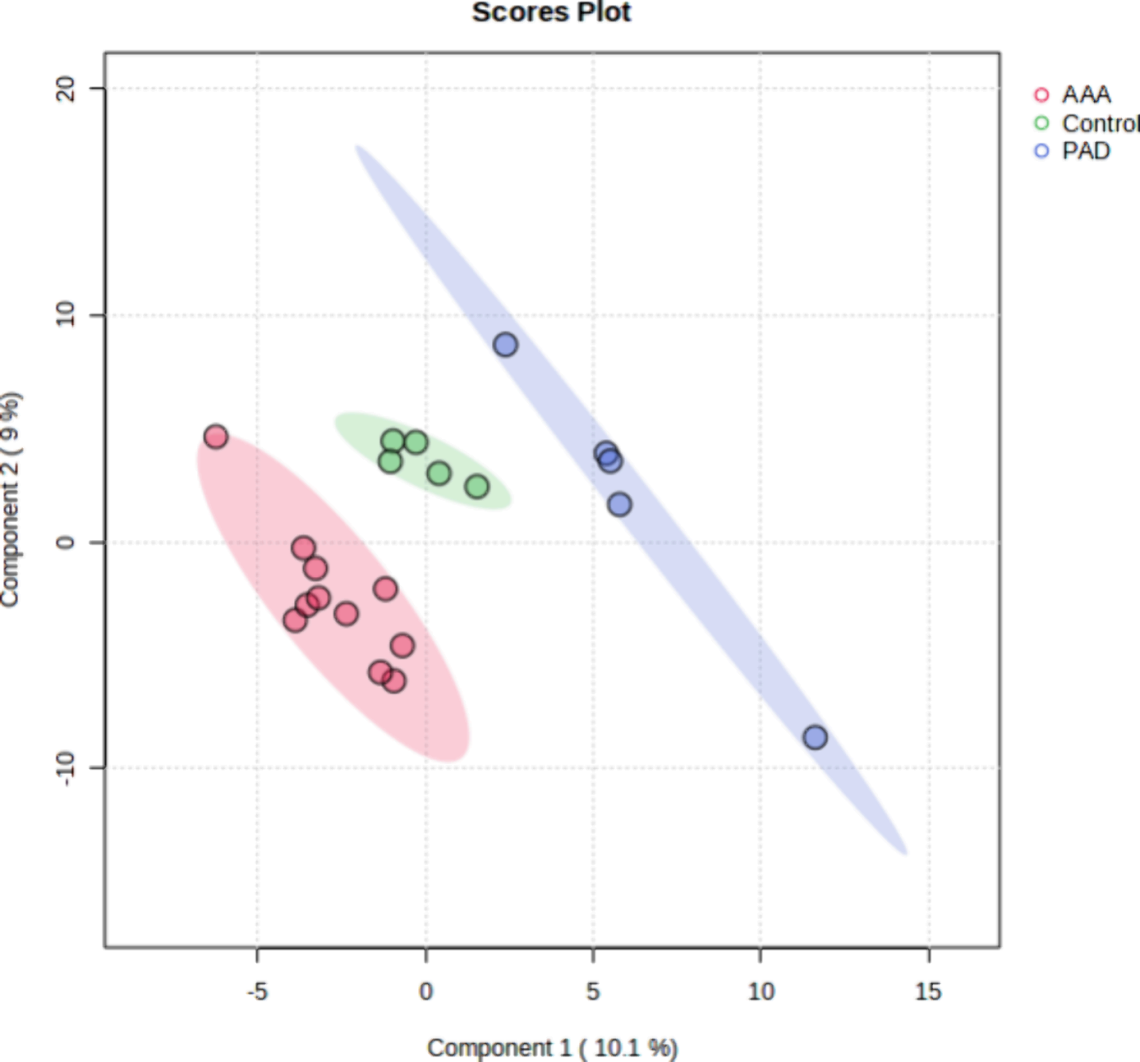
Two dimensional PLS-DA scores plot displaying the distribution of AAA, PAD, and Control plasma samples based on their lipidome fingerprint obtained in both positive and negative modes.

Finally, hierarchical clustering analysis of the plasma data set was used to create a heatmap of the top 50 lipid species with the lowest *q*-values. The dendrogram with two-dimensional hierarchical clustering of condition (AAA vs PAD vs Control) and variables (Figure 2) shows the 50 most important lipid species contributing to differentiate AAA and PAD from Control samples. It is possible to observe that in the first dimension, the top hierarchical dendrogram, the samples are clustered independently into three groups, AAA group (red), PAD group (blue), and the Control group (green). In the second dimension, there are two principal clusters. The first includes 13 PC [6 diacyl bearing polyunsaturated fatty acids (PUFA), 5 alkyl-acyl and 2 alkenyl-acyl], 5 alkenyl-acyl PE (also known as plasmenyl PE or PE plasmalogens), 2 PI, 2 PS, 4 CE, 1 CAR, 1 GM_3_, and 1 LPC that are all significantly reduced in the PAD group with a gradual increase of their relative abundances from AAA to control groups. The second cluster contains different sphingolipid and phospholipid species, which are significantly more abundant in both vascular diseases than in the control group, including 4 SM, 2 Cer, 1 HexCer, 3 PS, 4 PC (2 diacyl and 2 alkyl-acyl), 4 PE (3 diacyl and 1 alkenyl-acyl), 1 CAR, and 2 PI species.

**Figure 2:**
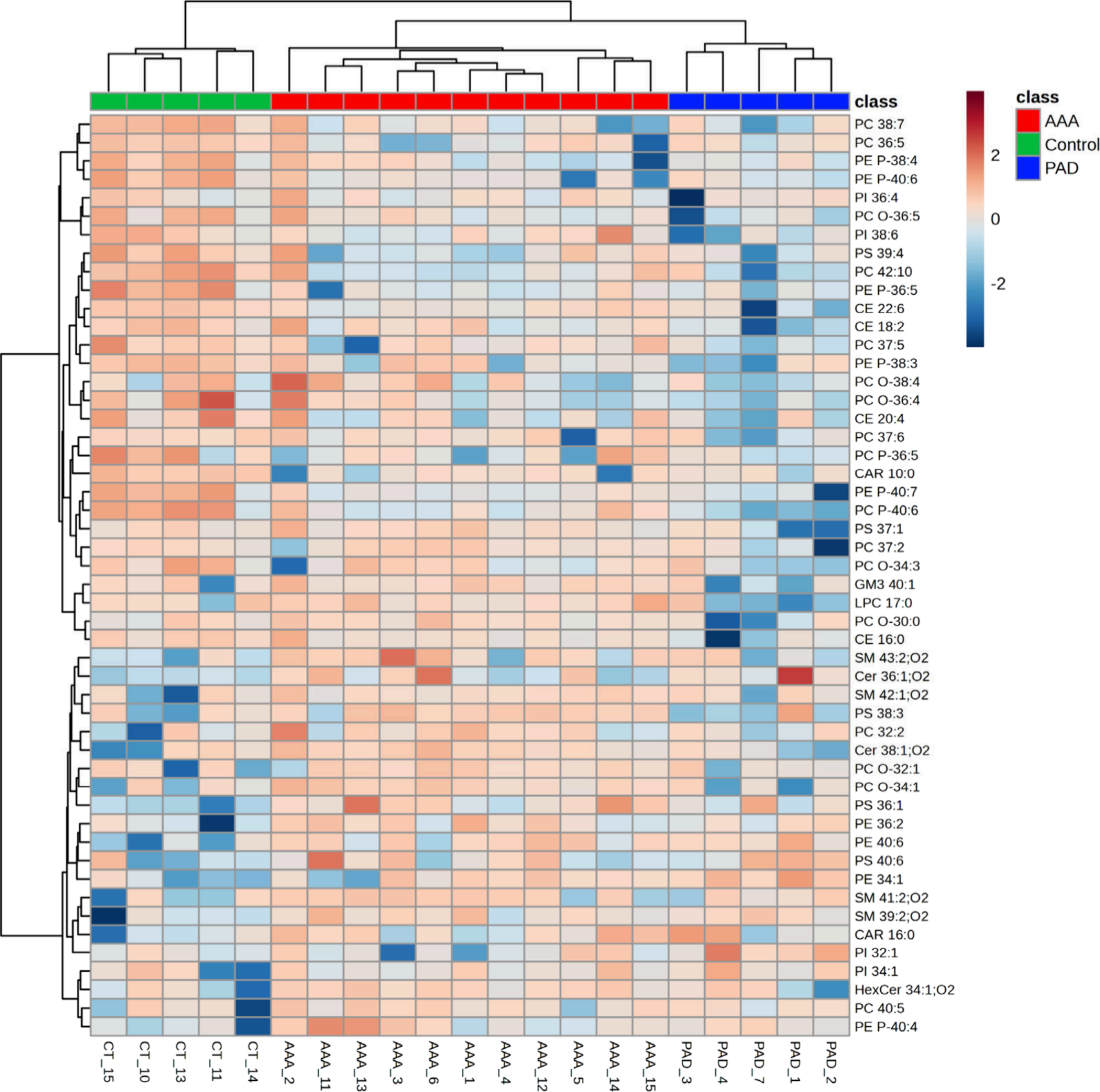
Two-dimensional hierarchical clustering heatmap of the 50 most discriminating lipid species of AAA, PAD, and Control groups. Relative abundance levels are shown on the red-yellow-blue scale, with the numbers indicating the fold difference from the overall mean. The red color of the tile indicates high abundance, and blue indicates low abundance. Null values were displayed in white. The clustering of the three groups is represented by a dendrogram at the top. The clustering of individual lipid molecular species is represented by the dendrogram on the left. CAR: fatty acylcarnitine, CE: cholesteryl ester, Cer: ceramide, GM3: ganglioside, HexCer: hexosylceramide, LPC: lyso PC, PC: diacyl phosphatidylcholine, PC-O: alkyl-acyl phosphatidylcholine, PC-P: alkenyl-acyl phosphatidylcholine, PE: diacyl phosphatidylethanolamine, PE-P: alkenyl-acyl phosphatidylethanolamine, PI: phosphatidylinositol, PS: phosphatidylserine, and SM: sphingomyelin.

## 4 Discussion

Considering that the application of clinical lipidomics in vascular diseases remains limited, there is little available information regarding lipid alterations in AAA and PAD with different matrices analyzed. Hence, we performed a comprehensive evaluation of the lipid molecular species from plasma samples of AAA and PAD patients that needed open repair surgery using a C18 RP-LC-MS/MS untargeted lipidomic approach. This work is a preliminary and exploratory study that serves as a basis lipidomic profile in AAA and PAD patients for future works that will be developed under the Horizon Europe VASCUL-AID project. The small sample size of this study must be disclosed as a significant limitation, potentially affecting the generalizability and statistical power of the findings, making it not only challenging to draw meaningful conclusions from the data but also may result in overfitting of the multivariate models. Nonetheless, the lipidomic analysis revealed several variations; thus, we proceeded with data interpretation and tried to attribute a reasonable meaning to the identified lipid changes. To have a broader perspective of the lipid profile of both vascular diseases, lipidomics data was initially analyzed by multivariate partial least-squares discriminant analysis (PLS-DA) (Figure 1). Interpretation of the PLS-DA score plot revealed that AAA and PAD have a different lipid profile when compared to the Control.

A hierarchical cluster analysis (HCA) was performed as well for visualization and interpretation of the variation of the lipid profile to provide insights into the specific lipid species that exhibit distinct modulation in both vascular diseases. Several lipids showed plasticity with the disease, namely, the phospholipids. PCs are the most abundant phospholipid in cellular membranes and lipoproteins^29^ and was the class that contributed the most to discriminate the three groups, comprising 17 species showing different behaviors. The diacyl and ether linked (both alkyl and alkenyl) PC species with PUFA, with equal or more than four double bonds, were found to have significantly reduced levels in PAD, followed by AAA. Meanwhile, mono- and diunsaturated PC species with diacyl and alkyl-acyl bonds, PC(32:2); PC­(O-32:1), and PC­(O-34:1), showed significantly higher abundances in both AAA and PAD, compared with controls. The decrease of levels of PC bearing PUFA might be ascribed to their degradation through lipid peroxidation processes,^30^ which occur in PAD and AAA due to high oxidative stress conditions associated with persistent inflammation of the artery wall.^31,32^ Also, the decrease in these species may compromise the availability of PUFA, which possess immunomodulatory effects.^33^ Therefore, the decrease in PUFA levels in patients with PAD and AAA, regarding the healthy control levels, may contribute to the increased inflammation associated with plaque formation and aneurysm growth, respectively.^12,34^ This finding aligns with the high levels of inflammatory markers found in these patients like C-reactive protein, IL-6, and TNF-α.^35,36^

After PC, PE is the second most abundant phospholipid in lipoproteins.^29^ In this study, 9 PE species were important to discriminate both vascular diseases, however showing dissimilar variations. Among these, the diacyl PE species PE(36:2), PE(40:6), and PE(34:1) exhibited higher levels in both vascular diseases than in controls. Diacyl PE species are valuable structural phospholipids of the cell membrane and are important players in the fusion/fission processes of the membrane.^37–40^ Thus, alterations in the levels of diacyl PE may lead to changes in the curvature of the cells, ultimately modifying the cell function. Additionally, diacyl PE are involved in autophagy and mitophagy processes by interacting with proteins from the autophagy-related protein 8 family.^41,42^ Moreover, increased levels of PE may lead to an enhanced autophagic flux, as reported for mammalian cells.^43^ This way, the higher levels of PE found in our study may be suggestive of membrane structure deformation and enhanced cellular autophagy leading to an imbalance of the bodỳs homeostasis.

On the other side, the ether-linked alkenyl (P-) species, are important players of the endogenous antioxidant defense system of the organism corresponding to 20% of total phospholipid content in mammals.^44^ The vascular wall inflammation during aneurysm and plaque formation, in AAA and PAD respectively, enhances an oxidative environment once it is associated with high reactive oxygen species production, leading to increased oxidative stress conditions.^31,32^ In this oxidative state, the PE plasmalogen species were expected to be increased in both vascular diseases, potentially acting as a countermeasure against heightened oxidative stress. However, plasmalogen PE species were found to have significantly lower relative abundances in both PAD and AAA (more pronounced in PAD), compared with the control group. Consequently, lower levels of these species may be associated with impairments or low capacity to avoid oxidative injuries and reactions,^45^ suggesting an inadequate function of the antioxidant defense system of these patients. In fact, endogenous antioxidant defenses [such as Cu/Zn and Mn superoxide dismutase, glutathione reductase and glutathione peroxidase,^46–48^ Paraoxonase-1,^49^ Nrf2/Heme-oxygenase 1 ^50^ and Glutathione-SH^50^] were shown to be dysregulated in AAA and PAD, as described in a recent review.^45^ Moreover, our results agree with the ones previously reported for atherosclerotic AAA and thoracic AAA (atherosclerotic and nonatherosclerotic), where the authors found reduced levels of PE plasmalogens in aortic tissue samples.^51^

The PS class contributed five species in the top 50 of HCA analysis. PS species showed opposite behaviors, with odd-chain PS(39:4) and PS(37:1) being significantly reduced mainly in PAD, followed by AAA, and even-chain PS(38:3), PS(36:1) and PS(40:6) increased in both vascular diseases when compared with controls. PS are important in signaling phospholipids in efferocytosis and apoptosis processes.^52^ Besides promoting cell clearance through “eat me” signals by PS exposure on the cell surface, PS may also exert influence on inflammation modulation. Some receptors of the CD300 receptor family are able to recognize PS molecules exposed on the outer leaflet of activated cell membranes.^53–55^ These receptors are responsible for the transduction of an inhibitory signal in mast cells, reducing the production of pro-inflammatory mediators,^56^ and for inhibiting dendritic cell-mediated antigen-specific T-cell responses.^57^ Nonetheless, the reason for the contrasting variations between odd- and even-chain PS species is unclear.

The PI class also made its way to the top lipid species in the HCA analysis, comprising four species. PI species bearing PUFA, PI(36:4), and PI(38:6), showed markedly lower abundances in PAD, followed by AAA, and higher levels of monounsaturated PI(32:1) and PI(34:1) species in both PAD and AAA, compared with controls. The reduction of PI species with PUFA, which goes hand in hand with the decrease of PC with esterified PUFA, may result from the degradation of these species by lipid peroxidation. Also, PI acts as an important mediator in signaling cascades^58^ thus, the decrease of these species further contributes to the heightened inflammatory state of the patients.

Sphingolipids were also considered to be important in this study. SM is commonly found in lipoproteins and plasma membranes, being extremely important to maintain the membrane structure. Four odd-chain SM species showed statistically significant differences between controls versus PAD and controls versus AAA, being increased in the plasma of patients with PAD and AAA. High levels of SM species have been linked with atherogenesis. The conversion of SM into Cer in atherogenic lipoproteins prompts lipoprotein aggregation and fusion, ultimately leading to the deposition of large lipoprotein aggregates similar to those found in extracellular regions of atherosclerotic lesions.^59^ Hence, our findings support an elevated risk of atherosclerotic events in AAA and PAD patients, which is in line with the atherosclerotic root of these vascular diseases. Cer can be synthesized through the hydrolysis of SM species thus an increase in SM levels should be accompanied by an increase of Cer.^60^ In this study, two Cer and one HexCer species also showed noticeably higher relative abundances in plasma samples of AAA and PAD, considering the healthy profile. This raise in Cer levels also supports the hypothesis of enhanced cellular apoptosis proposed for the observed variations of PS as they act as messengers in the activation of the apoptotic cascade.^61^

The neutral lipid class CE also contributed with four species to differentiate both vascular diseases and controls, CE(16:0), CE(18:2), CE(20:4), and CE(22:6), all being significantly decreased in PAD, gradually increasing their relative abundances from AAA to controls. Our results are in opposition with the ones previously reported in the literature where it has been identified high concentrations of CE, including CE(18:2), in tissue samples of the aneurysm wall in AAA^62^ and in perivascular adipose tissue of PAD patients.^63^

The use of statins may significantly modulate circulating lipid species and therefore constitute essential confounding or modulating factors in lipidomic studies. Published studies on the effect of statins on the lipid profile have reported the decrease in PC and PE (including diacyl and plasmalogen species), SM and Cer concentrations in both LDL fractions^64^ and plasma samples.^22^ Moreover, these species also changed their variation according with the type and dose of lipid-lowering therapy.^23,65^ Our results on PC, PE, SM and Cer subclasses are not in accordance with the aforementioned studies, which may suggest that the variation of these species might be disease-intrinsic rather than pharmacologically induced. These opposite findings emphasize the need for controlling or accounting for statins̀ intervention in patient cohorts requiring deeper research.

We studied AAA and PAD due to their common atherosclerotic root and aimed to ascertain any lipid metabolic alterations when compared with nondiseased controls (Figure 3). The knowledge acquired in this study will be applied in a future ongoing large-scale cohort under the VASCUL-AID project to perform untargeted lipidomic screening and targeted biomarker discovery for both AAA and PAD. In this study, we could not pinpoint any putative biomarker due to lack of comparable information and small sample size; however, the plasticity of the lipidome observed suggests a putative important adaptation of the lipid metabolism in these pathologies. Hence, more studies are needed to clarify the circulating lipidome of both vascular diseases with future works including (i) females also suffering from AAA and PAD to be more representative of the actual disease epidemiology and (ii) larger cohorts with balanced control groups to greatly improve lipid metabolism assessment and biomarker identification.

**Figure 3:**
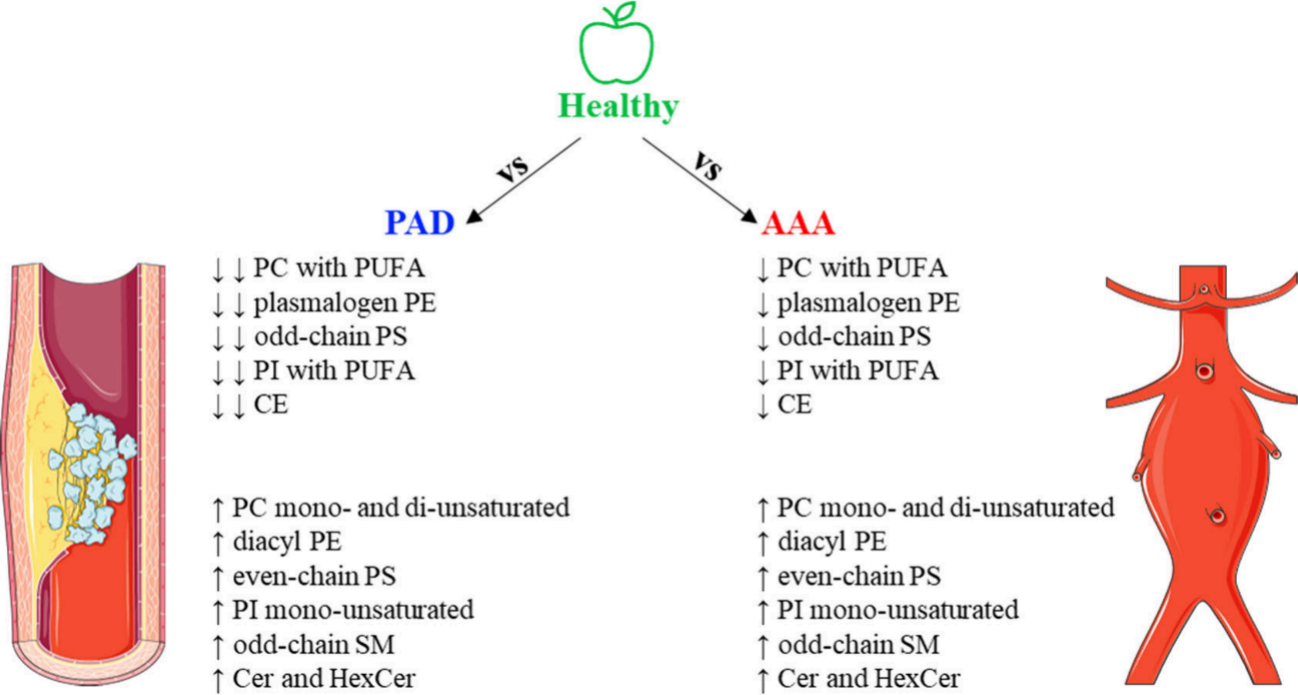
Lipid alterations determined in this study after lipidomic analysis of AAA and PAD patients compared with healthy controls. This figure was partly generated using Servier Medical Art, provided by Servier, licensed under a Creative Commons Attribution 4.0 unported license.

## 5 Conclusion

Plasma lipidomic analysis revealed that there is a different, although not deeply pronounced, lipid profile and lipid metabolism in AAA and PAD, compared with the controls. Phospholipids bearing PUFA, primarily PC, PE, and PI, showed a reduction in their relative abundances in AAA and PAD, while mono- and diunsaturated species were found to be upregulated. Additionally, AAA individuals showed reduced levels of PS and SM with odd chains. The results obtained in this study are either generally not in agreement with the ones reported in the literature (regarding phospholipids and neutral lipids) or do not have available information to compare with (regarding sphingolipids). This lack of comparability of the results might possibly be attributed to the low sample number, which is a limitation of this study; thus, it highlights the necessity for further studies in this matter, with larger cohorts, to better understand and clarify the underlying of pathophysiology-induced lipid modulation.

## Supplementary Material



## Data Availability

The data sets supporting this article have been uploaded as part of the supporting material. Data for this paper, including .raw data files are available at Science Data Bank, 2025 [2025-03-21] at https://cstr.cn/31253.11.sciencedb.22561. DOI: 10.57760/sciencedb.22561.

## Notes

Table S1. Demographic and clinical characteristics of patients with abdominal aortic aneurysm (AAA) and peripheral artery disease (PAD). Table S2. List of reagents and suppliers used in the study. All solvents were of high-performance liquid chromatography (HPLC) grade and were used without any additional purification. Table S3. Content of the internal standard mixture added to the lipid extract for the lipidomic analysis by RP-LC-MS/MS. Table S4. Lipid species identified in plasma samples of AAA, PAD and healthy controls by using C18-LC-MS/MS (mass error < 5 ppm). (XLSX)

## Notes

All procedures performed in studies involving human participants were in accordance with the ethical standards of the institutional and/or national research committee and with the 1964 Helsinki declaration and its later amendments or comparable ethical standards. Informed consent was obtained from all individual participants included in the study.
